# Mechanisms of transcriptional regulation in *Anopheles gambiae* revealed by allele specific expression

**DOI:** 10.1101/2023.11.22.568226

**Published:** 2023-11-22

**Authors:** Naomi A. Dyer, Eric R. Lucas, Sanjay C. Nagi, Daniel McDermott, Jon H. Brenas, Alistair Miles, Chris S. Clarkson, Henry D. Mawejje, Craig S. Wilding, Marc S. Halfon, Hasiba Asma, Eva Heinz, Martin J. Donnelly

**Affiliations:** 1 Department of Vector Biology, Liverpool School of Tropical Medicine, Pembroke Place, Liverpool, L3 5QA, UK; 2 Wellcome Sanger Institute, Wellcome Genome Campus, Hinxton, Cambridge, CB10 1SA, UK; 3 Infectious Diseases Research Collaboration (IDRC), Plot 2C Nakasero Hill Road, P.O.Box 7475, Kampala, Uganda; 4 School of Biological and Environmental Sciences, Liverpool John Moores University, Byrom Street, Liverpool, L3 3AF, UK; 5 Department of Biochemistry, Jacobs School of Medicine & Biomedical Sciences, University at Buffalo-State University of New York, 955 Main Street, Buffalo, New York 14203, USA; 6 Department of Clinical Sciences, Liverpool School of Tropical Medicine, Pembroke Place, Liverpool, L3 5QA, UK

**Keywords:** Allele, cis-regulation, transcript, insecticide, resistance

## Abstract

Malaria control relies on insecticides targeting the mosquito vector, but this is increasingly compromised by insecticide resistance, which can be achieved by elevated expression of detoxifying enzymes that metabolize the insecticide. In diploid organisms, gene expression is regulated both in *cis*, by regulatory sequences on the same chromosome, and by *trans* acting factors, affecting both alleles equally. Differing levels of transcription can be caused by mutations in *cis*-regulatory modules (CRM), but few of these have been identified in mosquitoes. We crossed bendiocarb resistant and susceptible *Anopheles gambiae* strains to identify *cis*-regulated genes that might be responsible for the resistant phenotype using RNAseq, and *cis*-regulatory module sequences controlling gene expression in insecticide resistance relevant tissues were predicted using machine learning. We found 115 genes showing allele specific expression in hybrids of insecticide susceptible and resistant strains, suggesting *cis* regulation is an important mechanism of gene expression regulation in *Anopheles gambiae.* The genes showing allele specific expression included a higher proportion of *Anopheles* specific genes on average younger than genes those with balanced allelic expression.

## Introduction

Malaria prevalence in Sub-Saharan Africa has reduced by 50% since 2000, primarily due to insecticide-based control of mosquito vectors (1). Recently, progress has stagnated (2), partly due to increasing levels of resistance against insecticides in mosquito populations (3). *Anopheles gambiae* is one of the dominant malaria vectors in Sub-Saharan Africa, the primary vector across most of Uganda (4) and the vector for which the largest resource of genome data is available, with 7275 genomes sequenced (5–8). A common cause of insecticide resistance is increased degradation of insecticides (termed metabolic resistance) (9) with overexpression of insecticide-metabolizing P450s repeatedly implicated (10–12). This can be caused by mutations in *cis*-regulatory regions regulating expression of metabolic resistance genes. In diploid organisms, such mutations only affect expression of the allele of the gene located on the same chromosome. Although some *trans* factors involved in metabolic resistance gene regulation in *Anopheles* are known (13, 14), few studies have identified genetic variation causing metabolic resistance (15–18). The multiallelic nature of metabolic insecticide resistance which can involve different mutations affecting the same gene in different populations, as well as the involvement of multiple genes, makes marker identification challenging as it limits the power of association studies unless very large sample sizes are used (15).

Despite the primary role of gene overexpression in metabolic resistance, only one *cis*-regulatory variant for resistance-linked differential expression has been identified in *Anopheles gambiae* (19), and markers for such variants are therefore absent in the current genetic marker panel for resistance [18]. Copy number variants (CNV) have been observed in *Anopheles gambiae* metabolic resistance gene clusters (20). For example, in *Anopheles coluzzi* copy number of *Cyp6AA1* is associated with deltamethrin resistance (21). The relative contribution of CNV and *cis*-regulation on *Anopheles* gene expression has not yet been determined.

Uganda sees a high burden of malaria; comprising 7.8% of all global cases in 2021 (22). To address this public health burden, the insecticide bendiocarb has been used for indoor residual spraying (IRS) to complement the distribution of long-lasting insecticidal nets (LLIN). Some resistance to bendiocarb in *An. gambiae* was observed in Nagongera (southeast Uganda) and Kihihi (southwest Uganda), with 83% and 70% mortality, respectively, to WHO bioassays with a diagnostic dose of 0.1% bendiocarb in 2014 prior to the IRS (23). The IRS campaign starting in December 2014 succeeded in reducing human biting rate, cases of malaria and test positivity rate in Nagongera (23), but the potential for further increases in resistance puts the long-term usefulness of bendiocarb into question.

Mosquitoes collected from Nagongera in 2014, which have moderate resistance to bendiocarb, showed significant differential expression of many genes compared to the susceptible Kisumu strain, including salivary gland protein encoding *D7r2* and *D7r4* genes as well as the detoxification associated genes *Gstd3* and *Cyp6m2* (24). Expression of *D7r4* was associated with a single-nucleotide polymorphism (SNP) in a non-coding transcript downstream of the D7 cassette (24).

**Figure 1:**
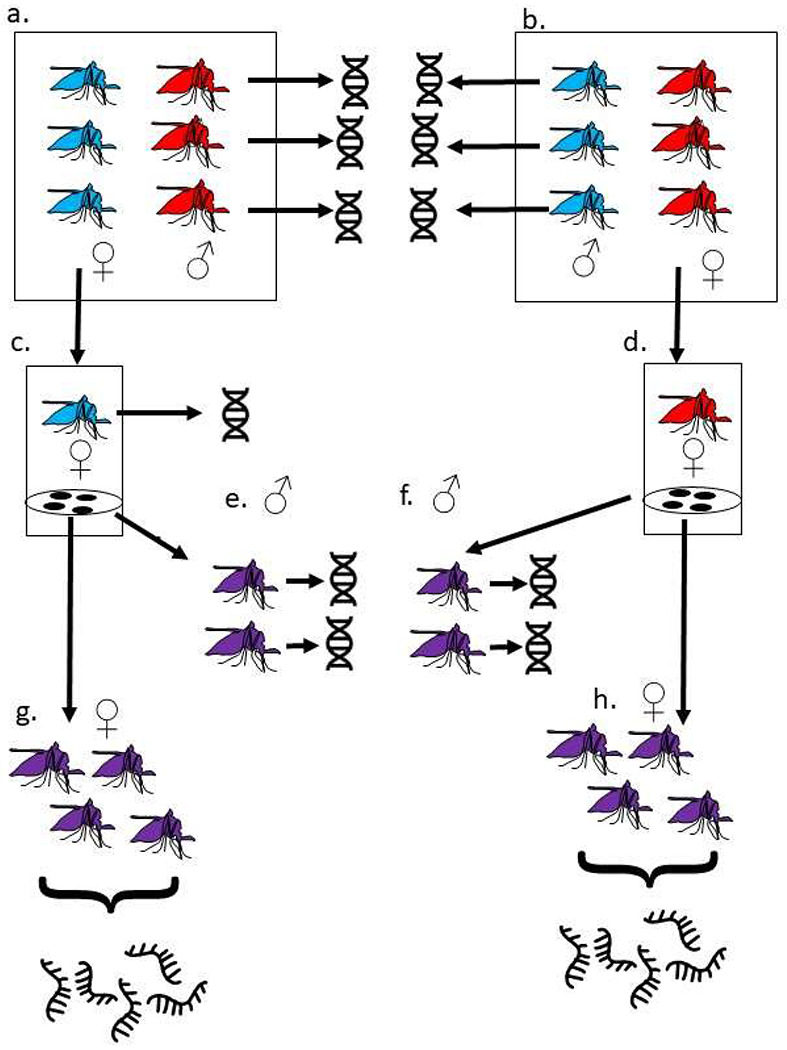
Crossing, DNA and RNA extraction schema a. 13 Kisumu females (blue) were crossed to 13 Nagongera males (red). Females mate only once. Following mating, genomic DNA from Nagongera males was extracted and sequenced. b. In the reciprocal cross, 13 Nagongera females (red) were crossed to 13 Kisumu males (blue). Following mating, genomic DNA was extracted and sequenced from Kisumu males. c. Individual mated Kisumu females were transferred to laying cups. Following egg laying, genomic DNA was extracted and sequenced from three of these d. Individual mated Nagongera females were transferred to laying cups. Following egg laying, genomic DNA was extracted and sequenced from three of these. e. The F1 progeny (purple) from the three Kisumu mothers sequenced at step c. were raised to adulthood. Genomic DNA was extracted and sequenced from individual males f. The F1 progeny (purple) from the three Nagongera mothers sequenced at step d. were raised to adulthood. Genomic DNA was extracted and sequenced from individual males g. and h. Female F1 from each of the six mothers were raised to adulthood. RNA was extracted and sequenced from a pool of ten F1 females three to five days after eclosion.

In diploid organisms, allele specific expression (ASE) provides strong evidence that genes may be under differential *cis*-regulatory control [19] (Figure 1). Using a method that has been applied to a variety of taxa [19–24] but not mosquitoes, we describe the identification of genes showing ASE in *An. gambiae* which potentially confer metabolic resistance. The challenges of applying this method that arise from mosquito biology and genome structure are discussed. In complementary work we predicted the sequences of some of the *cis* regulatory modules that may underlie the expression of genes involved in insecticide resistance using machine learning. Predictions included potential CRMs proximal to the genes showing ASE and genes that show consistent differential expression patterns in multiple resistant *Anopheles* strains, providing a starting point for future investigations into CRM variants during the evolution of insecticide resistance.

## Materials and Methods

### Strains and Crosses

Resting female mosquitoes were collected in Siwa Village, Nagongera, Tororo District in Eastern Uganda (0°46’12.0”N, 34°01’34.0”E) in March 2013 and allowed to lay eggs. 65 *Anopheles gambiae* (as ascertained by species ID PCR (25)) egg batches laid by the collected females were reared to larvae and combined to establish the Nagongera colony. All the colony founding mothers were screened for a known bendiocarb target site resistance mutation G119S in the *Ace1* gene using the TaqMan assay described in (26). No resistance associated variants were observed and full sequencing of the *Ace1* gene did not reveal any other potential target site resistance mutations. The colony was assayed using WHO diagnostic doses at F2 and was found to be highly resistant to DDT and deltamethrin, intermediate resistance to the carbamate insecticide bendiocarb and was highly resistant to pyrethroids and DDT. Bioassays using the WHO standard protocol (27, 28) with preexposure to 4% piperonyl butoxide (which inhibits P450 mediated metabolism) for one hour prior to bendiocarb exposure increased mortality compared to bendiocarb exposure alone, implying that bendiocarb resistance is metabolic rather than target site mediated.

A schematic of the experimental design is shown in figure 1. In brief, reciprocal crosses between Nagongera mosquitoes and an inbred insecticide susceptible colony (origin Kisumu, Kenya) were performed in cages as described previously (6). Reciprocal crosses each involved a total of 13 males and multiple females; since *Anopheles gambiae* are swarm maters, multiple males are required to induce mating. Following mating, females were transferred to individual cups for egg laying. F1 progeny of three females from each reciprocal cross were raised to adults under standard insectary conditions of 12 hours light 12 hours dark cycle, 26°C +/−2°C and 70% relative humidity, and fed on 10% sucrose solution.

### Sequencing

RNA was extracted from pools of ten female F1 progeny from each of the six crosses 3-5 days after eclosion using RNAqueous4PCR total RNA isolation kit (Invitrogen). RNA quality and quantity was checked to be adequate for library preparation (Agilent Bioanalyser and Qubit^®^ 2.0 Fluorometer). Total RNA libraries were prepared for Illumina paired-end indexed sequencing according to the Illumina TruSeq RNA sample preparation v2 guide (29). cDNA libraries were bar coded, pooled and sequenced using the Illumina HiSeq1500 platform, with 100bp paired end reads.

**Figure 2:**
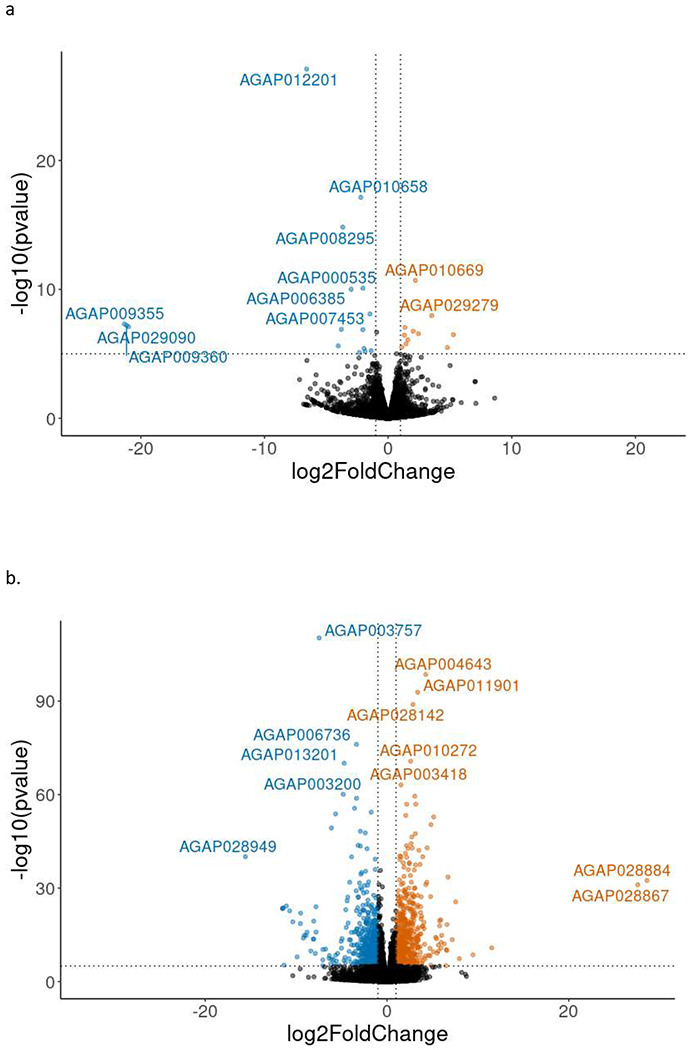
Reciprocal crosses show similar overall gene expression a. Volcano plot of log2 fold change against −log10 P value comparing gene expression in the F1 progeny of reciprocal crosses between Kisumu and Nagongera strains. Blue points indicate genes downregulated in progeny of Nagongera mothers compared to progeny of Kisumu mothers, orange points indicate genes upregulated in progeny of Nagongera mothers compared to progeny of Kisumu mothers. b. Volcano plot of log2 fold change against −log10 P value comparing gene expression between F1 progeny of Nagongera and Kisumu with the Kisumu parental strain. Blue points indicate genes downregulated in the cross progeny compared to Kisumu, orange points indicate genes upregulated in cross progeny of compared to Kisumu.

DNA was extracted using the Qiagen DNeasy Kit from the 6 individual mothers that laid viable eggs, all individual potential fathers that were alive following the cross, and individual male F1 siblings (Figure 2) and sequenced using the Illumina HiSeq 2000 as described previously (6).

### Genome analysis

Quality control of genomic sequences for mothers, potential fathers and F1 progeny, and matching of fathers to the progeny using Mendelian Error analysis was performed as described previously [6]. Briefly, for the autosomes for each cross where the mother’s genome sequence was of sufficient quality (K2, K4, K6 and B5), the per progeny sample Mendelian error was calculated for each possible parental pair of the known mother with all potential fathers using scikit-allel (30). The father in the parental pair that had the smallest median progeny sample Mendelian error on at least 3 autosomal arms was deemed to be the true father.

Scikit-allel [32] was used to analyze the genome of potential fathers including generating a consensus sequence of homozygous sites for Kisumu and Nagongera colonies, and calculating single nucleotide polymorphisms (SNPs) differing between the colonies.

### RNAseq analysis

Samples were compared using the rna-seq-pop snakemake workflow (31) which includes quality control of RNAseq reads using with FastQC (32) and MultiQC (33), read alignment to the *Anopheles gambiae* PEST reference genome (AgamP4, INSDC Assembly GCA_000005575.1, Feb 2006) using HISAT2 (34), principal components analysis to group samples by gene expression, differential expression analysis using Kallisto (35) with DESeq2 (36), variant calling using Freebayes version 1.3.2 (37) with ploidy 20 (as 10 diploid individuals were pooled in each sample), calculation of population diversity statistics with scikit-allel (30), and gene set enrichment analysis using GSEA (38). RNAseq data for the Kisumu parental colony, and Busia G28 deltamethrin selected colony (31) was compared with the cross progeny RNAseq data in this study.

### Analysis of allele specific expression (ASE)

For parent-based mapping, SNPs that were homozygous for different alleles in the parents were used to distinguish whether reads originated from a maternal or paternal allele: due to the pooling of 10 females for RNA sequencing it was not possible to use parental heterozygous SNPs. For sibling-based mapping, SNPs that distinguished maternal and paternal alleles on the autosomes were inferred from the genome sequences of the male siblings, using the following criteria: SNPs must be heterozygous and biallelic in all siblings where data was available. To eliminate potential low-quality SNPs, if 5 or more siblings had missing data at a SNP it was not used. Since F1 genome data was unphased, it was not possible to assign SNPs to either the Kisumu or Nagongera parent using this method. Analysis of F1 RNAseq data for ASE was performed using ASEReadCounter* (39), which is based on ASEReadCounter (40), using individual parent genomes where available for each cross to assign reads to parental alleles (options -vcf_mat, -vcf_pat). Where only sibling inferred SNPs were known, option -vcf_joind was used on the unphased inferred F1 VCF file.

The properties of SNPs inferred from parents and siblings were compared by inferring parental pseudogenomes and running ASEReadCounter* with both sets of SNPs. SNP level ASE was calculated for all SNPs with >=10 total counts. SNPs which were inferred from siblings and not from parents, which had >100 counts and ASE < .25 or > .75 were removed from the sibling inferred SNPs for subsequent analysis.

Due to overdispersion, RNAseq data is not binomially distributed but can be better described by a beta binomial distribution. The distribution of counts data and overdispersion parameter rho for the beta binomial distribution was calculated using the counts data for each cross, including the counts for the SNP with the maximum counts in each gene (with minimum 10 counts), using the R package glmmTMB (41), which uses maximum likelihood estimation to fit binomial and beta binomial model to the data for each cross. Models were compared using ANOVA.

To obtain a gene level measurement of ASE, the R package MBASED (42) was used to aggregate SNP level counts to gene level ASE by pseudo phasing SNPs in each gene such that the SNPs with the higher read count at each variable site are combined into the major allele, with frequency between 0.5 and 1. Only sites with at least ten counts were used. For SNPs inferred from the siblings of the RNAseq pool, MBASED was run assuming SNPs were not phased. Where phase was known from the parents, MBASED was run in both phased and non-phased mode to measure the impact on power to detect loci with ASE. In all cases the same seed (988482) was set prior to running 10^6^ simulations in which the total read count at each SNP position in each gene are kept constant, but reference allele counts are drawn from a null distribution beta binomial (mean 0.5 x total count, rho 0.038). The P value is the proportion of simulations in which the major allele frequency is greater than or equal to the observed major allele frequency. False discovery correction was applied with a nominal rate of 5% (43).

For SNPs inferred from parents, allelic count tables were additionally analyzed using Qleelic (39) with a binomial test of the null hypothesis of equal numbers of transcripts arising from maternal and paternal alleles. ASE was considered significant at p=0.05 following Bonferroni correction. Genes with fewer than ten read counts were excluded. As technical replicates were unavailable, an exploratory solution for correcting overdispersion was splitting reads into groups (pseudoreplicates K4 14 groups, K2 three groups, K6 nine groups, B5 nine groups). This produced a consistent quality correction constant (QCC) value of 1.56 for QCC correction. R packages UpSetR (44), stats and ggplot2 (45) were used for regressions and plots.

### Enrichment analysis

Hypergeometric tests for enrichment of gene ontology terms, Pfam protein domain enrichment, and KEGG pathways were implemented using python (46). The test set for each cross was the set of genes showing significant ASE following FDR correction, and the “universe set” was the set of genes for each cross that had at least ten reads containing SNPs distinguishing maternal and paternal alleles (for these genes ASE would have been detectable if present).

The evolutionary age of genes was taken from the Princeton Protein Orthology Database PPODv4_PTHR7-OrthoMCL (47), with age estimates based on Wagner parsimony. A gene age enrichment test was implemented using the age_enrichment.py script in ProteinHistorian (48), using option ‘-a ignore’ to ignore the proteins not present in the database. P values were obtained using a Mann Whitney U test for difference in overall age distribution between sets and Fisher’s exact test for difference in fraction of proteins in each age in the set.

The null hypothesis, the proportion of genes showing significant allelic imbalance of expression does not differ between autosome arm, was tested using a two-sided Fisher’s exact test (for those crosses where the counts were too high for efficient computation P value was simulated using 2000 replicates).

To assess selective sweeps, lists of genes showing ASE were compared to a database of regions under selection as defined by H_12_ signal (49) both across the range of *Anopheles gambiae* and restricted to Uganda using a custom Python script (https://github.com/azurillandfriend/sweeps_ASE.git).

### Prediction of *cis*-regulatory modules

*Anopheles* cis-regulatory modules (CRM) were predicted using SCRMshaw s (50–53). Briefly, *Drosophila* CRM which drive gene expression in the tissue of interest were downloaded from the Redfly database (v9.6.0, database updated 02/01/2023) (54, 55),with max size 2000. Only non-overlapping sequences (>100bp) were included. Syntenous regions from related *Drosophila* species (putatively containing the equivalent CRM) were added to the training data set using liftOver at https://genome.ucsc.edu/cgi-bin/hgLiftOver (56) as described in (51). SCRMshaw was trained using this augmented training set and a 10x bigger set of non-CRM non-exonic regions. Repeats in CRM, non-CRM and the target *Anopheles gambiae* PEST genome were masked using repeat masker (57). Existing training sets for adult peripheral nervous system, embryonic and larval excretory, and embryonic/ larval Malpighian tubules were downloaded from GitHub (https://github.com/HalfonLab/dmel_training_sets). These were compared with the 2272 Transposase Tn5 hypersensitive sites identified by Ruiz et al (58).

## Results and Discussion

### Crosses

Three females from each reciprocal cross between the Kisumu and Nagongera strains laid viable eggs (Figure 1, Table 1). For four crosses the father was identified by minimizing median Mendelian error (Supplementary Table 1, but for the other two crosses the mother failed sequencing quality control and none of the sequenced putative fathers were a good match. We presume the true fathers for these crosses died during the experiment, precluding extraction of good quality DNA.

RNAseq statistics for the pooled F1 females from each of these six crosses are shown in Table 1. The number of reads recommended for 60% power to detect ASE at 1.5 fold is 500 per gene [37], which for the 13,796 annotated genes in *Anopheles gambiae* would require around 6.9x10^6^ reads. Five crosses exceeded this value with one cross, K2, having slightly fewer (6.4 x10^6^reads). The proportion of reads mapping to the *Anopheles gambiae* genome was similar for all crosses (mean 87.0%, standard deviation 1.1%).

### Between pools gene expression comparison

Comparison between reciprocal crosses revealed similar gene expression in the F1 progeny, with just 38 genes significantly downregulated and 38 genes significantly upregulated in the F1 between reciprocal crosses at P_adj_ ≤ 0.05 (Figure 2a). When comparing only F1 crosses in PCA, B1 seemed to be an outlier in terms of expression (Supplementary figure 1a). However, when compared with other colonies, the F1 progeny formed a distinct group (Supplementary figure 1b).

F1 gene expression differed from the Kisumu (parental) colony, with a total of 1362 genes upregulated and 1265 genes downregulated in the F1 compared to the Kisumu colony (Padj ≤ 0.001) (Figure 2b). RNAseq data was unavailable for the Nagongera colony, which no longer exists. Gene set enrichment analysis of the differentially expressed genes indicated that genes with Gene Ontology (GO) terms associated with odorant binding, olfactory receptor activity, sensory perception of smell, response to stimulus, detection of chemical stimulus involved in sensory perception of smell, structural constituent of cuticle and signal transduction were upregulated in the F1 compared to Kisumu, whereas those with GO terms associated with translation, the ribosome, mitochondrion, structural component of cuticle, and serine type endopeptidase activity were downregulated (all at P adjusted 0.05). KEGG pathway analysis showed significant upregulation of ribosome, citrate cycle (TCA cycle) and oxidative phosphorylation in the F1 compared to Kisumu.

Although we did not perform RNAseq on the Nagongera colony, a previous study compared gene expression in mosquitoes collected from the same location in 2014 (one year later than the colony founders) with the susceptible Kisumu colony using microarrays (24). We compared the genes that show significant differential expression between Kisumu and F1 in RNAseq (4848 genes) and the earlier microarrays’ 3363 genes. A total of 1320 genes were on both these lists, including *D7r4* which was upregulated in both Nagongera and the cross compared to Kisumu. However, only in 645/1320 genes was the direction of fold change the same in both comparisons highlighting the difficulty of comparing microarray and RNAseq expression studies, that gene expression may have changed both during the 2013-2014 period as well as during selection of the Nagongera colony, and possible over and underdominance of expression in the F1.

### Selection of SNPs to detect ASE

Detection of ASE relies on sufficient SNP differing between the parents (59). The number of SNPs differing between maternal and paternal genomes varied (Table 2). For crosses B1 and B3, with parent genomes unavailable, we instead attempted to use parental colony consensus sequences for mapping. However, the number of SNPs differing in Nagongera and Kisumu consensus sequences (Table 3) was insufficient for ASE analysis. The low number of differing SNPs may be due to barriers to recombination imposed by the genome structure of *Anopheles gambiae*, which limit the extent to which colonies become inbred, with heterozygosity persisting even after many generations of inbreeding (60). This contrasts with *Drosophila* where colony genotypes have been used effectively for inference of ASE (61, 62). Since the mosquitoes were pooled prior to RNA extraction, inferring SNPs from the RNAseq data to detect ASE is problematic, since most SNPs are heterozygous in one or both parents, resulting in a mixture of homozygous and heterozygous SNPs in the progeny, and we would not know whether to test for deviation from 1:1 expression or 3:1. To increase the number of SNPs to detect ASE in these pooled samples we therefore used the siblings of the RNA sequenced F1 to infer which SNPs were homozygous but different between the parents (opposite homozygous SNPs). For a Mendelian inherited SNP, opposite homozygous SNP in parents should result in all progeny having heterozygous biallelic SNP. If one or both parents were heterozygous (biallelic) then 50% of the progeny are expected to be heterozygous, and the probability of all the progeny being heterozygous in this situation is given by the binomial probability density function where p=0.5, the number of trials (per SNP) is the number of progeny and the number of “successes” is also the number of progeny. The number of progeny and SNPs per chromosome arm where all progeny were heterozygous is given in Supplementary Table 2. Almost all sequenced siblings were male, the heterogametic sex in *An. gambiae*, so it was not possible to infer SNPs on the X chromosome using this method.

**Figure 3:**
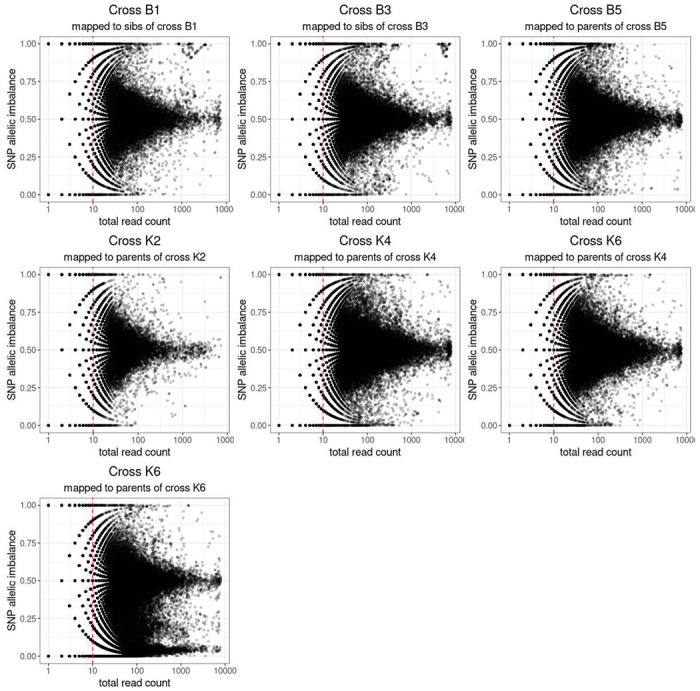
Allele balance in progeny of crosses between strains Plot of total read count against allelic imbalance at each SNP. Cross and source for the SNPs used to map and count reads are indicated at the top of each plot. Cross K6 is shown with allelic imbalance inferred using SNPs from both the parents of cross K4 and for the initially assumed parents of cross K6.

The suitability of these SNPs to detect ASE was first tested by comparing the SNPs inferred from the siblings to those inferred from the parents, where parental genotypes were known (Supplementary table 3). Most SNPs were shared between the parents and siblings, with only a small fraction (<7.5%) unique to the siblings. The total number of usable SNPs from the siblings was lower than those in the parents: a reduction of between 6% and 16%. Per SNP ASE was calculated as counts at reference SNP/counts at reference SNP plus counts at alternative SNP for the sibling SNPs, and as counts at maternal SNP/ counts at maternal plus paternal SNP for parent SNPs. The mean per SNP ASE for SNPs with at least ten reads counted remained between 0.49 and 0.51 confirming that the SNPs are likely to be heterozygous in the F1 and that mapping bias is not a problem for this dataset. The standard deviation of per SNP ASE increased very slightly (from 0.12-0.14 to 0.13 to 0.15). For crosses B1 and B3, the per SNP ASE was in line with the other crosses with mean 0.5 and standard deviation 0.13 and 0.15 respectively. However, when the per SNP ASE was plotted against the total counts at each SNP, a cluster of SNPs a with high ASE and expression was seen for the sibling but not parent inferred SNPs (Figure 3). SNPs in crosses K2, K4 and B5 that were unique to siblings and with high ASE and high expression were therefore removed from the analysis for crosses B1 and B3 before inferring per gene ASE.

### ASE inference

For the four crosses where the parent was known, per gene ASE was calculated as reads mapping to maternal genome/total reads mapped at that gene. In addition the major allele frequency at each gene was calculated for all crosses. Read count data were fitted to binomial and beta binomial models. ANOVA indicated that the beta binomial model was a better fit to the data (P < 2.2e-16). The dispersion parameter phi(disp) for the beta binomial distribution of counts data for crosses B1, B3, B5, K2, and K4 was estimated at 25.8, giving a rho 1/(1+phi(disp)) of 0.038. Per gene ASE statistics for all crosses are shown in Table 4. Due to variations in parental genome and sequencing depth the power to detect ASE varies between crosses, so it is not possible to infer whether the proportion of genes with ASE is different between the crosses. Three crosses showed similar numbers of genes with maternal or paternal ASE, whereas cross K6 surprisingly seemed to show extreme paternal ASE. Using SNPs from F1 male siblings to infer ASE also showed a larger number of genes with ASE in cross K6 than the other crosses. To investigate whether K6 shows a very different pattern of ASE to the other crosses, or a sampling error occurred so that the RNA sequenced samples were not from the same cross as the parents and siblings. We therefore checked the effect of having selected the wrong SNPs to infer allelic imbalance. SNPs inferred from parents of the other crosses were used to infer allelic imbalance for cross K6. If incorrect SNPs are used ASE is artificially inflated due to counting reads at sites where some or all the progeny are homozygous. When the SNPs from the parents of cross K4 were used to infer allelic counts for cross K6, the mean imbalance was shifted back to 0.5, suggesting that these SNPs are more suitable than the erroneously inferred parents (figure 3, Supplementary table 4). Furthermore, when SNPs called from the RNAseq data were compared, the progeny of cross K4 and K6 were extremely genetically similar and clustered closely in PCA for all chromosomal arms, suggesting they may have hatched from two egg batches from the same cross. The analysis for cross K6 was therefore repeated using SNPs from K4 parents.

**Figure 4:**
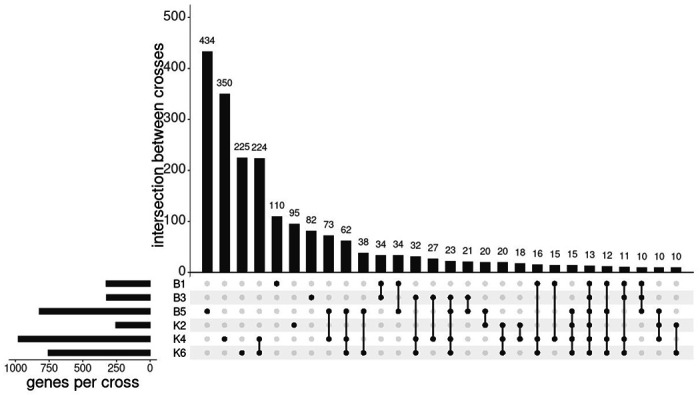
Intersection of genes showing ASE between crosses UpSet plot with the number of genes showing ASE for each cross and the intersection of these genes between crosses. Only the first 28 sets of overlaps are shown.

Genes showing ASE showed some overlap between the different crosses (Figure 4). At the most conservative estimate, 13 genes showed ASE in all crosses. This exceeds the overlap expected by random genes showing allelic imbalance, as in 100,000 simulations of randomly drawing the observed number of significant genes from the set genes where SNPs were available to detect ASE in all crosses, the maximum expected overlap was 1. Most genes showing ASE were unique to individual crosses (Figure 4), but there were many genes showing ASE in combinations of multiple crosses that may also be under consistent differential *cis*-regulation between Tororo and Nagongera colonies, Table 5 shows the 115 genes with significant ASE in at least 4 out of the 6 crosses. Directionality of ASE was inferred for crosses B5, K2, K4 and K6 (Supplementary Table 7).

**Figure 5:**
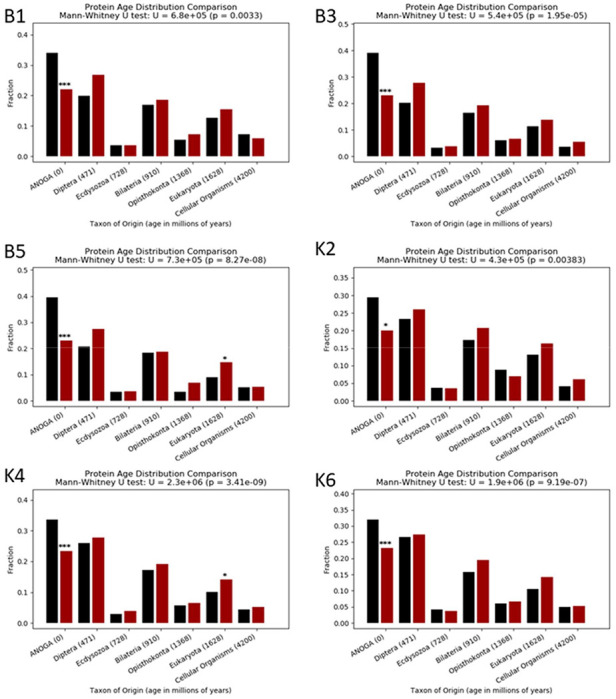
Ages of genes showing ASE or not showing ASE bar plots comparing the age of genes which showed ASE or did not in the progeny of six crosses between Nagongera and Kisumu strains, using the Wagner parsimony method. Fisher’s exact test P values for the difference in fraction of genes in each age between genes showing ASE (red bars) and genes not showing ASE (black bars) are displayed above the bars; *: P<0.05, ***:P<=0.001.

Analysis of gene ages that showed or did not show ASE revealed an enrichment of younger, *Anopheles* specific genes showing significant ASE (Figure 5 and Supplementary Table 5). The same trend was seen in all crosses.

**Figure 6:**
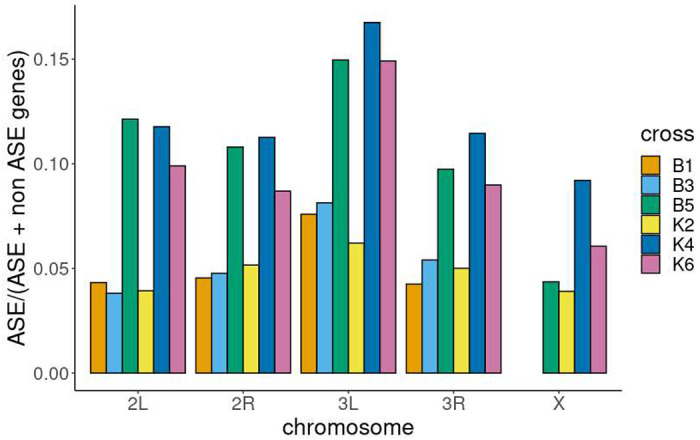
proportion of genes showing ASE versus genes with sufficient SNPs to detect ASE along each chromosome arm Bar plot of the proportion of genes showing allele specific expression/ total genes containing suitable SNPs to detect ASE per chromosome arm. Colours indicate the different crosses. X chromosomal SNPs were not available for crosses B1 and B3

Genes showing ASE showed an unequal distribution along chromosomal arms compared to genes not showing ASE (Supplementary table 6, Figure 6, Supplementary figure 2). This trend was significant for all crosses except for K2. The trend was for slight enrichment on 3L. For the 4 crosses where parents were available, this was also compared with the X chromosome, which had a lower proportion of ASE/total detectable genes (Figure 6, Supplementary figure 2). ANOVA indicated both chromosome and cross explained the variance in the proportion of ASE/total detectable genes per chromosome arm (P < 0.05).

### Copy number variation

True ASE is caused by *cis*-regulation, but copy number variation of the expressed gene could lead to apparent ASE if there are different numbers of gene copies containing the SNPs used to count reads; e.g. if two copies of a duplicated gene with total of three copies bear one SNP and the third the other SNP, apparent ASE would be inferred without differential *cis*-regulation. We therefore checked for CNV in the parental and F1 sibling genomes at the genes that showed ASE in at least four out of six crosses, and in the genes which contained sufficient SNPs to infer allelic imbalance but expression appeared to be in balance. Copy number variation was deemed possible if any of the parents and F1 siblings from crosses B5, K2, K4 or K6 (total 63 individuals) had a copy number call other than 2 on the autosomes. As ASE could not be inferred on the X chromosome for crosses B1 and B3 we excluded it from the analysis. A total of 60/114 (53%) autosomal genes showing ASE contained possible CNV. For genes that did not show ASE and had available SNPs to infer ASE in all crosses, 481/1333 (36%) had possible CNV. A two-sided Fisher’s exact test rejected the hypothesis that the odds ratio is 1 (odds ratio 2.0, P=0.0006). This suggests that for some genes CNV may be contributing to the observed ASE. The number of individuals with a possible CNV in each gene varied between the genes (Table 5)

### Overlaps

The list of 115 genes showing ASE in at least four crosses was compared with published gene expression data. For all other available data, it is not known to what extent the expression is *cis* or *trans* regulated, meaning that lack of consistency between the datasets may be due to the absence of *trans* regulated genes in the ASE data.

Genes showing ASE in at least four crosses were compared with genes with consistently high median fold change in a meta-analysis of 35 experiments comparing RNAseq data between *Anopheles gambiae* s.l. and *Anopheles funestus* strains (46), and with genes showing significant fold change with consistent directionality in microarray data comparing susceptible and resistant populations of *Anopheles coluzzi* (63). For the RNAseq metadata set, the genes with the top 5% of median fold changes between susceptible and resistant populations (429/8599 total genes in the dataset), six genes were also present in the ASE gene set. These were AGAP001251 (*Eupolytin*), AGAP008218 (*Cyp6Z2*) and AGAP012296 (*Cyp9J5*), AGAP011068 (*Aldose reductase*), AGAP003583 (*L-iditol 2-dehydrogenase*) and AGAP008331 (*WD repeat-containing protein 59*). There was no overlap with the 40 genes showing in microarray.

Of the gene families previously implicated in metabolic resistance, we observed four P450s with ASE in four of the six crosses: *Cyp12F3* (AGAP008019), *Cyp12F2* (AGAP008020), *Cyp6Z2* (AGAP008212) and *Cyp9J5* (AGAP012296).

The D7 protein family has previously been implicated in bendiocarb resistance in Uganda (24). AGAP008282 (*D7r2*) showed significant ASE in two crosses (detectable in 4) and *D7r4* in none (detectable in 3). Other *D7r* genes, *D7r1* (AGAP008284), *D7r5* (AGAP008280) also showed significant ASE in two crosses, *D7L1, D7L2* and *D7r3* in one cross.

Gene set enrichment analysis did not reveal consistent enrichment of any particular gene ontology term amongst genes showing ASE across the progeny of the crosses.

We finally asked whether genes showing ASE were more likely to be in a genomic region that has undergone a recent selective sweep. Out of 115 genes showing ASE in at least 4/6 crosses, 13 were in a swept region, whereas 103 of the 1333 genes showing no evidence of ASE in any of the 6 crosses were in swept regions. A two sided Fisher’s exact test did not reject the hypothesis that the odds ratio is 1 (odds ratio 1.5, P=0.2), suggesting that genes showing ASE are no more likely to be in a swept region than genes that do not.

### CRM prediction

*Drosophila* CRM for adult midgut, adult Malpighian tubules, larval midgut and legs were used as training data from SCRMshawHD to predict *Anopheles* CRM operating in the same tissues, together with previously developed training sets for the adult peripheral nervous system, embryonic and larval excretory system and embryonic/ larval Malpighian tubules (https://github.com/HalfonLab/dmel_training_sets). These tissues were selected based on previous studies examining the tissue specific expression of genes involved in insecticide resistance with roles including detoxification and cuticular resistance (64–69). Training CRM sets used for the first time in this study are shown in table 6, with full sequences at https://github.com/azurillandfriend/traning_sets_IR.git. Oenocyte (FBbt:00004995), cuticle (FBbt:00004970) and adult epidermis (FBbt:00005401) CRM could not be used as training data due to insufficient known CRM, highlighting the need for more research to identify CRM in these tissues. The top scoring 250 predictions for each training set and method were combined, producing a total of 4122 unique CRM predictions. 62 of these predicted CRMs were flanked by a gene showing ASE in at least 4/6 crosses (Table 7). In total, CRM were predicted for 33 of the 115 genes showing ASE in at least 4/6 crosses. 211 predicted CRM were flanked by a gene showing consistently high median fold change between resistant and susceptible strains (Supplementary table 8). CRM were predicted for a total of 141 of the 429 genes in this set. The predicted CRM were also compared with a published dataset of Tn5 transposase sensitive sites in the adult midgut (58, 70). 50 of the predicted CRMs overlapped with Tn5 transposase sensitive sites identified as *cis*-regulatory elements by Ruiz *et al* (58), flanking a total of 48 genes. The vast majority of these CRMs (47) were previously predicted by Kazemian (71) despite the different training sets. The CRMs identified in these predictions provide a starting point for future studies to examine genetic variation in *Anopheles* populations with different insecticide resistance phenotypes, and for validation using reporter assays.

## Conclusions

Sample pooling, while a cost-effective solution for bulk RNAseq of small samples, does limit the power of experiments targeting the study of ASE; in future, to maximize the power to detect ASE, individual samples should be used. RNAseq on whole mosquitoes may have masked tissue specific ASE. This is likely to increase the number of genes showing ASE, since pleiotropic effects may limit the potential for genes to up or down regulated in all tissues simultaneously but permit the evolution of tissue specific regulation (72). Future studies should target specific tissues of interest. Despite these limitations, we were able to detect genes showing ASE in pooled RNA from whole mosquitoes in crosses between *Anopheles* strains with different carbamate resistance phenotypes, indicating different *cis*-regulation patterns between the strains. Whilst we detected some genes previously implicated in insecticide resistance, there was no consistent enrichment of these or any particular gene ontology term amongst genes showing ASE. This probably indicates that the strains have undergone extensive *cis*-regulatory divergence, affecting both genes involved in insecticide resistance but also genes involved in many other functions. Comparing ASE in progeny of crosses between multiple insecticide resistance and susceptible strains, together with examining gene expression in the parental strains, would enable a more comprehensive survey of the *cis* versus *trans* regulation of insecticide resistance genes. The bias towards younger, *Anopheles* specific genes showing ASE in the F1 suggests there may be a higher degree of *cis*-regulatory divergence between the parental strains for younger genes. It was possible to computationally predict some CRM involved in tissue specific expression, including potential CRM for genes showing ASE and genes previously implicated in insecticide resistance. This was hampered by the lack of good quality training data for the tissues thought to be most relevant to insecticide resistance in adult mosquitoes, highlighting the need for future experimental CRM discovery.

## Supplementary Material

Supplement 1

Supplement 2

## Figures and Tables

**Table 1:** Crosses between Kisumu and Nagongera and RNAseq summary statistics

RNAseq sample name	Cross name	Mother origin	Mother-Sanger code	Father origin	Father – Sanger code	Total reads counted (F1 RNAseq)	Percent of reads mapping to *Anopheles gambiae* PEST genome
Wilding_1	B1	Nagongera	failed QC	Kisumu	unknown	18,496,263	88.6%
Wilding_2	B3	Nagongera	failed QC	Kisumu	unknown	38,526,717	87.1%
Wilding_3	B5	Nagongera	AC0382-C	Kisumu	AC0416-C	28,969,694	87.7%
Wilding_4	K2	Kisumu	AC0300-C	Nagongera	AC0406-C	6,400,460	85.5%
Wilding_5	K4	Kisumu	AC0317-C	Nagongera	AC0398-C	46,229,505	86.3%
Wilding_6	K6	Kisumu	AC0334-C	Nagongera	AC0398-C	29,491,773	86.9%

**Table 2:** Number of SNPs in exonic regions differing between the parents that could be used to detect ASE

Cross	2L	2R	3L	3R	X	Y / other	total
K2	13849	30134	19375	20877	15543	0	99778
K4	33139	35048	19074	22405	15250	0	124916
K6	30303	30811	14534	17801	15601	2	109052
B5	29627	36203	20865	25414	10539	1	122649

2L, 2R, 3L, 3R and X are the chromosome arms. The numbers are the number of SNPs in exonic regions that are homozygous in the parents of each cross but differ between the parents. These sites should be heterozygous in all cross progeny.

**Table 3:** Number of SNPs differing between Kisumu and Nagongera colony consensus sequences

Chromosome	SNPs
2L	2575
2R	13507
3L	6216
3R	2949
X	10552
Total	35799

“SNPs” shows the number of SNPs differing across all sites in the consensus genome sequences of Kisumu and Nagongera colonies

**Table 4:** Number of genes showing ASE in each cross

Cross	Genes showing ASE	Total genes with SNP(s) and sufficient counts to detect ASE
B1	327	6486
B3	324	6065
B5	823	7537
K2	255	5185
K4	979	8166
K6	757	7852
All (intersection)	13	2468

ASE detected with FDR of 5%

**Table 5:** Genes showing ASE in at least four crosses

ID	chr	Gene Name	Description	overlap	het ASE	Freq CNV	Gene ASE in cross			
							B5	K2	K4	K6
AGAP001002	X		Toll protein	4	0	NA	0.00	1.00	0.96	0.98
AGAP001190	2R		female reproductive tract protease GLEANR 896	6	0	18	0.92	0.06	0.22	0.12
AGAP001243	2R			4	4	NA	0.38	0.03	0.28	0.28
AGAP001251	2R		eupolytin	5	0	3	0.24	NA	0.83	0.92
AGAP001356	2R	ACE1	Acetylcholinesterase	5	2	15	0.52	0.35	0.22	0.27
AGAP001365	2R			4	0	15	0.57	0.44	0.38	0.32
AGAP001594	2R			4	4	1	0.45	0.63	0.71	0.75
AGAP001726	2R			4	0	4	0.75	0.47	0.23	0.31
AGAP001971	2R		Polyubiquitin-C	4	5	11	0.55	0.59	0.64	0.69
AGAP002008	2R			4	0	NA	0.17	0.80	0.76	0.79
AGAP002058	2R		beta-galactosidase	4	0	3	0.14	0.83	0.82	0.93
AGAP002728	2R		Isocitrate dehydrogenase [NAD] subunit, mitochondrial	4	0	5	0.60	0.38	0.33	0.36
AGAP003364	2R			4	0	NA	0.61	0.44	0.33	0.36
AGAP003417	2R			4	0	NA	0.41	0.53	0.67	0.62
AGAP003444	2R			4	2	22	0.27	0.62	0.84	0.78
AGAP003474	2R			6	0	NA	0.49	0.63	0.61	0.63
AGAP003514	2R			4	3	NA	0.33	0.84	0.72	0.66
AGAP003525	2R		CCR4-NOT transcription complex, subunit 2	4	2	2	0.49	0.72	0.66	0.64
AGAP003583	2R		L-iditol 2-dehydrogenase	4	0	8	0.30	NA	0.32	0.06
AGAP003657	2R			4	0	NA	0.08	0.76	0.67	0.64
AGAP003663	2R		ATP-dependent RNA helicase DBP2	4	3	NA	0.43	0.62	0.60	0.58
AGAP003669	2R		homeobox protein MSX	4	2	NA	0.33	0.58	0.68	0.64
AGAP003686	2R			4	0	1	0.40	0.58	0.62	0.66
AGAP003701	2R		zinc finger protein 593 homolog	4	0	62	NA	NA	0.14	0.34
AGAP003722	2R	ANXB10C	annexin B10C	4	3	6	NA	NA	0.73	0.71
AGAP003728	2R		Uncharacterized methyltransferase WBSCR22	4	0	1	0.34	0.53	0.72	0.69
AGAP003731	2R		vacuolar protein sorting-associated protein 37B	4	4	1	0.49	0.57	0.74	0.77
AGAP003802	2R			4	3	NA	0.37	0.62	0.60	0.53
AGAP003861	2R		Peptide deformylase	4	0	1	0.34	0.61	0.73	0.73
AGAP004102	2R			4	0	1	0.36	0.49	0.65	0.66
AGAP004109	2R		Xaa-Pro aminopeptidase	6	6	NA	0.74	0.28	0.37	0.40
AGAP004110	2R			6	3	NA	0.79	0.23	0.23	0.25
AGAP004113	2R	mRpL55	39S ribosomal protein L55, mitochondrial	5	0	4	0.75	0.34	0.36	0.36
AGAP004133	2R			4	1	35	0.35	0.97	0.63	0.75
AGAP004233	2R		transcription factor IIIB 90 kDa subunit	6	1	NA	0.19	0.76	0.74	0.76
AGAP004534	2R		cathepsin B precursor	4	0	NA	0.87	NA	0.21	0.23
AGAP004562	2R		MFS transporter, PCFT/HCP family, solute carrier family 46 (folate transporter)	4	0	NA	0.86	0.49	0.14	0.27
AGAP004790	2L		Up-regulated during skeletal muscle growth 5 homolog	4	0	4	0.05	NA	0.79	0.79
AGAP004891	2L		metallophosphoester ase domain-containing protein 1	4	2	NA	NA	NA	0.70	0.65
AGAP005012	2L		galactokinase	4	0	30	0.92	NA	0.21	0.18
AGAP005243	2L			4	2	NA	0.37	0.67	0.47	0.44
AGAP005548	2L		alpha-crystallin B chain	4	1	1	0.67	0.96	0.75	0.82
AGAP006690	2L		BTB (POZ) domain containing 9	4	0	NA	0.68	NA	0.62	0.70
AGAP006756	2L			4	0	NA	0.57	NA	0.74	0.68
AGAP006775	2L		glucosyl/glucuronosyl transferases	4	0	NA	0.87	NA	0.80	0.86
AGAP007159	2L		alpha-crystallin B chain	4	3	4	0.27	NA	0.17	0.13
AGAP007402	2L			4	1	33	0.61	0.90	0.87	0.86
AGAP007407	2L	CTLMA4	C-type lectin (CTL) - mannose binding	4	0	2	0.47	0.07	0.27	0.26
AGAP007413	2L			4	1	NA	0.56	0.76	0.64	0.60
AGAP007484	2L		Sugar transporter ERD6-like 4	5	0	NA	0.86	0.58	0.17	0.30
AGAP008019	3R	CYP12F3	cytochrome P450	4	0	14	0.78	NA	0.12	0.09
AGAP008020	3R	CYP12F2	cytochrome P450	4	0	46	0.87	0.47	0.21	0.27
AGAP008122	3R		U6 snRNA-associated Sm-like protein LSm7	4	2	NA	0.61	NA	0.40	0.40
AGAP008218	3R	CYP6Z2	cytochrome P450	4	1	1	0.89	NA	0.66	0.47
AGAP008325	3R		U3 small nucleolar RNA-associated protein 23	4	0	NA	0.58	0.29	0.27	0.30
AGAP008331	3R		WD repeat-containing protein 59	4	0	56	0.70	0.77	0.03	0.03
AGAP008551	3R		mothers against decapentaplegic homolog 1	4	0	NA	0.55	0.53	0.90	0.80
AGAP008849	3R		D-3-phosphoglycerate dehydrogenase	4	5	NA	0.54	0.48	0.48	0.46
AGAP009251	3R			4	2	26	0.86	NA	0.20	0.13
AGAP009418	3R			4	2	63	0.17	0.91	0.48	0.77
AGAP009518	3R			4	1	NA	0.47	0.41	0.45	0.41
AGAP009527	3R			5	0	6	NA	0.38	0.37	0.34
AGAP009585	3R			5	0	41	0.23	NA	0.07	0.28
AGAP009782	3R		methylosome protein 50	4	3	10	0.28	0.55	1.00	1.00
AGAP009791	3R	Nep1	neprilysin, neutral endopeptidase 1	4	0	NA	0.32	0.66	0.69	0.63
AGAP010658	3L			5	0	1	0.30	NA	0.94	0.85
AGAP010814	3L	TEP6	thioester-containing protein 6	5	5	14	0.73	0.30	0.68	0.64
AGAP010873	3L		Cytosolic Fe-S cluster assembly factor NUBP2 homolog	5	3	NA	0.56	0.06	0.19	0.15
AGAP011065	3L			4	3	63	0.65	0.51	0.76	0.65
AGAP011091	3L			4	0	1	0.51	NA	0.70	0.72
AGAP011155	3L		dual specificity phosphatase	4	0	NA	0.77	0.27	0.32	0.32
AGAP011202	3L			4	0	14	0.48	0.58	0.60	0.60
AGAP011379	3L	GPRFZ1	frizzled receptor	4	4	NA	0.75	0.51	0.37	0.38
AGAP011499	3L			4	4	63	0.49	0.56	0.66	0.69
AGAP011616	3L			4	0	NA	0.40	0.55	0.69	0.78
AGAP011750	3L		alpha-N-acetylglucosaminidase	4	0	NA	0.70	0.36	0.28	0.35
AGAP011783	3L	CLIPA13	CLIP-domain serine protease	5	0	NA	0.38	0.91	0.73	0.74
AGAP011784	3L			4	0	NA	0.83	0.53	0.16	0.53
AGAP011792	3L	CLIPA7	CLIP-domain serine protease	4	0	NA	0.63	0.41	0.36	0.38
AGAP012008	3L			5	3	NA	0.83	0.23	0.78	0.86
AGAP012053	3L		alpha,alpha-trehalase	6	5	NA	0.68	0.33	0.42	0.38
AGAP012100	3L	RpS26	40S ribosomal protein S26	4	0	5	0.20	0.79	0.77	0.71
AGAP012118	3L		FGFR1 oncogene partner 2-like protein	5	4	NA	0.54	0.47	0.63	0.59
AGAP012126	3L		lysophospholipase II	5	4	NA	0.95	0.26	0.09	0.11
AGAP012130	3L	ATN	allatotropin	6	4	NA	0.89	0.03	0.05	0.07
AGAP012135	3L		26S proteasome regulatory subunit N8	5	3	8	0.35	0.55	0.65	0.64
AGAP012148	3L		Mitogen-activated protein kinase	6	6	NA	0.71	0.18	0.15	0.18
AGAP012152	3L			4	2	NA	0.72	0.29	0.24	0.27
AGAP012154	3L		solute carrier family 15 member 1	6	5	NA	0.75	0.18	0.29	0.25
AGAP012188	3L		ubiquinol-cytochrome c reductase subunit 7	5	3	4	0.68	0.23	0.30	0.42
AGAP012189	3L		abhydrolase domain-containing protein 12	6	3	NA	0.88	0.19	0.09	0.11
AGAP012217	3L		sterol O-acyltransferase	6	4	21	0.80	0.41	0.78	0.71
AGAP012219	3L	Med1	mediator of RNA polymerase II transcription subunit 1	5	4	1	0.41	0.40	0.32	0.30
AGAP012296	3L	CYP9J5	cytochrome P450	4	2	36	0.28	0.64	NA	NA
AGAP012305	3L		Muscle LIM protein at 84B	4	4	NA	0.37	0.66	NA	NA
AGAP012314	3L			4	4	2	0.23	NA	0.86	0.86
AGAP012984	2R		Protease m1 zinc metalloprotease	4	0	2	0.69	0.33	0.30	0.31
AGAP012996	2R		juvenile hormone-inducible protein	5	0	NA	0.38	0.63	0.59	0.60
AGAP013039	2R			4	0	NA	0.84	0.17	0.26	0.38
AGAP013188	2R	APN4	aminopeptidase 4	5	4	61	0.31	0.86	0.62	0.58
AGAP013255	2R	APN3	aminopeptidase N1	4	3	62	0.34	0.93	0.37	0.36
AGAP013359	2R			4	0	1	0.95	0.06	0.04	0.14
AGAP013393	2R	APN2	aminopeptidase N1	4	3	62	0.50	0.17	0.26	0.35
AGAP028064	3R			4	1	NA	0.25	NA	0.88	0.85
AGAP028157	3L			4	0	36	0.32	0.75	0.84	0.78
AGAP028209	3R			4	1	1	0.38	0.41	0.35	0.39
AGAP028542	3L			6	0	6	1.00	0.21	0.00	0.00
AGAP029122	3L			4	5	NA	0.40	0.49	0.68	0.62
AGAP029263	3L			4	4	4	0.76	0.17	0.25	0.29
AGAP029287	3L			5	4	NA	0.89	0.00	0.06	0.07
AGAP029362	2L			4	0	NA	0.19	0.71	0.80	0.63
AGAP029417	3R			4	1	37	0.91	0.06	0.12	0.12
AGAP029453	3R			5	4	NA	0.54	0.63	0.75	0.69
AGAP029526	2L			4	2	2	0.61	1.00	0.60	0.57
AGAP029597	3L			6	2	NA	0.96	0.15	0.03	0.03

Chrom: chromosome

ID: Vectorbase gene ID

Overlap: the number of crosses that showed significant allelic imbalance of expression of the gene, when using SNPs from the siblings to infer allelic expression.

Expression biased to the maternal allele is highlighted in red and expression biased to the paternal allele highlighted in blue.

Freq CNV is how many of the 63 parents and siblings with CNV calls (crosses K2, K4, K6 and B5) had a possible CNV covering that gene.

**Table 6:** training CRM

Tissue	Anatomy ontology term	Date of search	Total Number of CRM <2000bp in Redfly at time of search	Number of CRM meeting criteria (non overlapping)
Adult Midgut	FBbt:00003138	12/12/22	12	11
Larval Midgut	FBbt:00005825	12/12/22	16	15
Adult Malpighian Tubules	FBbt:00005725	12/12/22	10	9
Leg	FBbt:00004640	12/12/22	35	28
Antennae	FBbt:00004511	24/04/23	25	20
Oenocyte	FBbt:00004995	25/04/23	3	2

**Table 7:** CRM predictions for genes that showed ASE in at least 4/6 crosses

chrom	Flanking gene 1	Name flanking gene 1	flanking_gene_2	Name flanking gene 2	CRM length	models	score	CRM start	CRM_end	training_sets
2L	AGAP005012		AGAP005013		500	imm	7.12	8246250	8246750	Adult Malpighian tubules
2L	AGAP005012		AGAP005013		500	imm	4.85	8288000	8288500	Adult midgut
2L	AGAP005244		AGAP005243		500	hexmcd	26.41	12797250	12797750	malpig.mapping1
2L	AGAP006690		AGAP006689		500	pac	0.39	36723500	36724000	Larval midgut
2L	AGAP006755	snRNP-U1-70K	AGAP006756		500	imm	17.54	37866500	37867000	emb-larv_excretory,malpig.mapping1
2R	AGAP001194		AGAP001190		500	imm	6.75	1190000	1190500	Adult Malpighian tubules
2R	AGAP001356	ACE1	AGAP001355		500	hexmcd	12.68	3467250	3467750	legs
2R	AGAP013039		AGAP001990		500	pac	0.35	13217500	13218000	Adult Malpighian tubules
2R	AGAP013039		AGAP001990		750	hexmcd, pac	11.86	13224000	13224750	Adult Malpighian tubules
2R	AGAP002007		AGAP002008		500	pac	0.36	13703500	13704000	Adult midgut
2R	AGAP002007		AGAP002008		500	pac	0.41	13710000	13710500	Adult pns
2R	AGAP002007		AGAP002008		500	pac	0.42	13711750	13712250	Adult pns
2R	AGAP002007		AGAP002008		500	hexmcd, imm, pac	16.10	13717000	13717500	Adult midgut, antenna
2R	AGAP002007		AGAP002008		500	pac	0.44	13721000	13721500	emb-larv excretory
2R	AGAP002007		AGAP002008		500	hexmcd, imm	14.42	13727000	13727500	legs
2R	AGAP002007		AGAP002008		500	hexmcd	13.10	13773750	13774250	Larval midgut
2R	AGAP002008		AGAP002007		500	pac	0.41	13804500	13805000	legs
2R	AGAP002008		AGAP002009		500	pac	0.40	13843500	13844000	legs
2R	AGAP003473		AGAP003474		500	hexmcd	10.65	38103750	38104250	Adult Malpighian tubules
2R	AGAP003663		AGAP013457		500	imm	5.17	41330500	41331000	Adult pns
2R	AGAP003669		AGAP003667		500	pac	0.39	41423000	41423500	Adult pns
2R	AGAP003669		AGAP003670		500	hexmcd	11.76	41493500	41494000	Adult pns
2R	AGAP003727		AGAP003728		500	hexmcd	12.09	42615500	42616000	Adult pns
2R	AGAP013081		AGAP004110		500	pac	0.33	50078500	50079000	Adult midgut
2R	AGAP004112		AGAP004113	mRpL55	500	pac	0.39	50141750	50142250	antenna
2R	AGAP004112		AGAP004113	mRpL55	500	pac	0.40	50151250	50151750	legs
2R	AGAP004112		AGAP004113	mRpL55	500	hexmcd	12.06	50160250	50160750	antenna
2R	AGAP004232		AGAP004233		500	pac	0.38	52891500	52892000	Larval midgut
2R	AGAP004232		AGAP004233		500	pac	0.34	52894750	52895250	Adult Malpighian tubules
2R	AGAP004232		AGAP004233		500	pac	0.34	52921250	52921750	Adult midgut
3L	AGAP010813	TEP18	AGAP010814	TEP6	500	hexmcd	13.38	11182250	11182750	Adult pns
3L	AGAP011064		AGAP011065		500	imm	5.20	16137250	16137750	Adult pns
3L	AGAP011379	GPRFZ1	AGAP011378		500	pac	0.36	22779500	22780000	Larval midgut
3L	AGAP011379	GPRFZ1	AGAP011378		500	pac	0.37	22796500	22797000	Larval midgut
3L	AGAP012008		AGAP012007		500	imm	10.24	36201750	36202250	Larval midgut
3L	AGAP012026		AGAP028157		500	imm	10.72	36626250	36626750	legs
3L	AGAP012026		AGAP028157		500	imm	10.90	36645500	36646000	antenna
3L	AGAP012026		AGAP028157		500	hexmcd	29.43	36652000	36652500	emb-larv excretory, legs, malpig.mapping1
3L	AGAP029616		AGAP012053		500	hexmcd	12.69	36968750	36969250	legs
3L	AGAP029616		AGAP012053		500	hexmcd, imm	29.45	37009250	37009750	emb-larv_excretory, legs, malpig.mapping1
3L	AGAP029122		AGAP012093		750	hexmcd, imm	35.98	37603750	37604500	emb-larv excretory, malpig.mapping1
3L	AGAP029122		AGAP012093		500	imm	11.19	37608750	37609250	antenna
3L	AGAP029612		AGAP012118		500	hexmcd	26.81	37824750	37825250	emb-larv_excretory,malpig.mapping1
3L	AGAP029612		AGAP012118		500	imm	9.59	37830500	37831000	Larval midgut
3L	AGAP012313		AGAP012314		500	pac	0.45	40131000	40131500	malpig.mapping1
3L	AGAP012313		AGAP012314		500	hexmcd	11.57	40135250	40135750	antenna
3L	AGAP012313		AGAP012314		500	pac	0.44	40137750	40138250	malpig.mapping1
3L	AGAP012313		AGAP012314		500	pac	0.37	40140500	40141000	Larval midgut
3R	AGAP028064		AGAP028015		500	pac	0.38	333000	333500	Larval midgut
3R	AGAP008019	CYP12F3	AGAP008018	CYP12F4	500	imm	8.26	4322500	4323000	Adult midgut
3R	AGAP008551		AGAP008552	CYP4H27	500	imm	11.67	12539500	12540000	Adult midgut
3R	AGAP008553		AGAP029453		500	pac	0.40	12582000	12582500	Adult pns
3R	AGAP029453		AGAP008557		500	pac	0.37	12662750	12663250	Larval midgut
3R	AGAP029417		AGAP009416		500	pac	0.40	32426500	32427000	Adult pns
3R	AGAP009418		AGAP009417		500	pac	0.36	32603250	32603750	Adult midgut
3R	AGAP009518		AGAP029410		500	imm	17.77	35145750	35146250	emb-larv_excretory, malpig.mapping1
3R	AGAP009518		AGAP029410		500	pac	0.40	35148250	35148750	antenna
3R	AGAP009522		AGAP009527		500	hexmcd	20.04	35340750	35341250	Adult midgut
3R	AGAP009522		AGAP009527		500	hexmcd, imm	11.01	35348500	35349000	Larval midgut
3R	AGAP009527		AGAP009522		500	pac	0.37	35364000	35364500	Larval midgut
3R	AGAP009790	CPAP3-B	AGAP009791	Nep1	500	hexmcd	17.04	43310250	43310750	Adult midgut
3R	AGAP009790	CPAP3-B	AGAP009791	Nep1	500	imm	4.90	43317000	43317500	Adult midgut

The positions of the 62 predicted CRMs for genes that showed ASE in at least 4/6 crosses. The two closest genes flanking each CRM are shown, with the flanking gene that shoed ASE highlighted in green

## Data Availability

RNAseq data for all crosses and read counts per SNP are available at Gene Expression Omnibus accession GSE241768. Potential fathers sequencing data are available at European nucleotide archive (accession numbers in Supplementary Table 9). Sequencing data from siblings from crosses B1 and B3 are at European nucleotide (accession numbers in supplementary Table 10) All other nucleotide sequences used are already publicly available in the Phase 3 release of the *Anopheles gambiae* 1000 genomes project, at accession PRJEB42254.
